# Deep Neural Network for Differentiation of Brain Tumor Tissue Displayed by Confocal Laser Endomicroscopy

**DOI:** 10.3389/fonc.2021.668273

**Published:** 2021-05-11

**Authors:** Andreas Ziebart, Denis Stadniczuk, Veronika Roos, Miriam Ratliff, Andreas von Deimling, Daniel Hänggi, Frederik Enders

**Affiliations:** ^1^ Department of Neurosurgery, University Hospital Mannheim, Medical Faculty Mannheim, University of Heidelberg, Mannheim, Germany; ^2^ Department of Software Engineering, Clevertech Inc., New York, NY, United States; ^3^ Department of Neuropathology, University Hospital Heidelberg, and CCU Neuropathology, DKFZ, Heidelberg, Germany; ^4^ Department of Neurosurgery, Medical Faculty, Heinrich-Heine-University Düsseldorf, Düsseldorf, Germany

**Keywords:** confocal laser endomicroscopy, deep neural network, machine learning, brain tumor, fluorescein sodium, image analysis

## Abstract

**Background:**

Reliable on site classification of resected tumor specimens remains a challenge. Implementation of high-resolution confocal laser endoscopic techniques (CLEs) during fluorescence-guided brain tumor surgery is a new tool for intraoperative tumor tissue visualization. To overcome observer dependent errors, we aimed to predict tumor type by applying a deep learning model to image data obtained by CLE.

**Methods:**

Human brain tumor specimens from 25 patients with brain metastasis, glioblastoma, and meningioma were evaluated within this study. In addition to routine histopathological analysis, tissue samples were stained with fluorescein *ex vivo* and analyzed with CLE. We trained two convolutional neural networks and built a predictive level for the outputs.

**Results:**

Multiple CLE images were obtained from each specimen with a total number of 13,972 fluorescein based images. Test accuracy of 90.9% was achieved after applying a two-class prediction for glioblastomas and brain metastases with an area under the curve (AUC) value of 0.92. For three class predictions, our model achieved a ratio of correct predicted label of 85.8% in the test set, which was confirmed with five-fold cross validation, without definition of confidence. Applying a confidence rate of 0.999 increased the prediction accuracy to 98.6% when images with substantial artifacts were excluded before the analysis. 36.3% of total images met the output criteria.

**Conclusions:**

We trained a residual network model that allows automated, on site analysis of resected tumor specimens based on CLE image datasets. Further *in vivo* studies are required to assess the clinical benefit CLE can have.

## Introduction

Intraoperative diagnosis continues to be an essential tool during neurosurgical procedures; nevertheless it remains challenging. Sites frequently lack the possibility of immediate pathologist’s interaction and therefore require time for delivery and processing, and the frozen sections are subject to sampling errors (1–3).

Confocal laser endomicroscopy (CLE) can be used for intraoperative visualization in fluorescence guided surgery and offers cellular resolution. CLE has already been successfully applied to facilitate surgery for head and neck neoplasms and urological surgical procedures (4–6). Furthermore, it is used in gastroenterology for diagnosis of Barrett’s esophagus and colorectal lesions among others (7, 8). Moreover, the application of CLE showed promising results in thyroid surgery and bronchoscopy including biopsy collection for interstitial lung disease (9, 10).

Since its start, efforts were made matching the features of CLE images to the histopathological sections; however, the transfer of classical neuropathological characteristics of common brain pathologies to CLE images is limited (11, 12). Until recently, application of CLE to central nervous tumors was mostly limited to preclinical studies. The first clinical trials show promising results for CLE to become part of routine intraoperative tumor diagnosis and might help detect tumor remnants in neurosurgery (12, 13). By extending the resection borders at a cellular level, this technique has the promising potential to protect normal brain tissue. However, there is only one optical fluorescence filter available and fluorescein, the most thoroughly investigated fluorescence dye, is not routinely used for brain tumor surgery. Further, for optimal utilization a high amount of intraoperatively collected image data needs to be directly analyzed.

Deep learning models reduce expenditure of time and interobserver-biased evaluation of intricate structures (14). The neural network models are successful where labeled data is available. Multiple recent applications of computer vision and medical imaging have shown cutting-edge performance (15, 16).

We hypothesized that a model integrating features from conventional CLE using a machine learning approach could diagnose tumor origin and identify specific features relevant to the entity.

## Material and Methods

### Patient Cohort

The training and validation cohort consisted of patients with histologically confirmed glioblastoma, brain metastasis, or meningioma WHO grade I, treated at the neurosurgical department of the University Hospital of Mannheim. The current 2016 WHO classification of brain tumors was used. All patients whose histopathological analyses did not confirm glioblastoma WHO grade IV, brain metastasis, or meningioma WHO grade I were excluded (n = 10). Our final patient cohort included 25 patients (median age 56.3 years; 19–82.2 years, male/female: 9/16).

### Tumor Tissue Processing

Brain tumor tissue was collected from 25 adult patients who underwent resection of a brain tumor at the University Hospital Mannheim. Fresh tumor samples were immediately processed and split into sister specimens. Tissue pieces (2–5 mm in diameter) were incubated in fluorescein solution for 30 min (Fluorescein 10%, ALCON Pharma GmbH, Freiburg, Germany, final concentration 0.1 mg/ml, diluted with Ringer’s solution) and subsequently washed with Ringer’s solution three times for 2 min. As a positive control adjacent tumor specimens were stained with Nuclear Green for 1 min (Abcam, Cambridge, United Kingdom, 50 µM final concentration, diluted with Ringer’s solution), incubated for 5 min, and washed once for 5 min with Ringer’s solution. All working steps were performed at room temperature. After immediate CLE imaging, tissue samples were analyzed by the Department of Neuropathology of the University Hospital Heidelberg corresponding to routine diagnostics including H&E, immunostaining and in certain cases methylation analysis. Pathology confirmed newly diagnosed glioblastoma in eight patients, brain metastasis in eight patients (six patients presented with non-small cell lung cancer and two with breast cancer) and meningioma WHO grade I in nine patients.

### Confocal Laser Endomicroscopy

For CLE imaging the OptiScan System, model CIS-CZM-B-CP (SN R&D 4011-05; Zeiss, Oberkochen, Germany) including a sterile sheath was used. Briefly summarized, light is transmitted by a laser source (488 nm wavelength) through an optical fiber to the hand held scanner probe. The focal signal is detected by exciting the fluorescent dye by laser light. Latter is converted into a digital pattern depending on the amount of emitted fluorescence. This pattern can be transformed into a grayscale image parallel to the lens’ plane with adjustable focus depth. Images were acquired immediately after staining. The probe was fixed, and specimens were applied on the lens to minimize motion induced artifacts.

Images were created with 1920 × 1080 pixels and were obtained with 0.75 frames per second and a focus depth of 0 to 120 µm. In a second step, we removed data sets with low imaging quality affecting the whole image due to technical issues, such as blood and motion artifacts or out of focus scanning, from further evaluation.

### Image Preprocessing and Convolutional Neural Networks

A convolutional neural network (CNN) is a widely adopted network of machine-learning algorithms to process multiple arrays such as images which detect local conjunctions using convolutional and pooling layers before fully connected layers are following. The given hierarchy in images leads to lower-level features composing higher-level features in a deep neural network. Residual convolutional (ResNet) and Inception networks (InceptionNet) have been applied in clinical classification problems. A ResNet18 and Inception network was constructed with the Pytorch framework (https://pytorch.org/) written in python as a tensor and dynamic network. ResNet18 has 17 convolutional layers and contains batch normalization and identity mappings, additionally. InceptionNet-v3 was used consisting of seven inception blocks, pooling layers and normalization layers. We used four fully connected layers for both networks. The last fully connected layer gives a classification according to the global features connected from all local features. We used negative log likelihood as loss function and log softmax after the fully connected layers. Stochastic gradient decent was selected for optimization with a learning rate of 0.01 and a momentum of 0.5. We applied data augmentation techniques to enlarge the database with analogical but not identical data. Techniques, such as vertical and horizontal flipping methods, were applied to inflate the size of the training dataset and to reduce overfitting. Images were scaled down to 960 × 540 pixels, and sections of 400 × 400 pixels were chosen randomly. The probability of horizontal and vertical flipping was set to 0.5. Images were fed in batches with a batch size of 32 for ResNet and 16 for InceptionNet, respectively. Eventually, the enhanced training data was fed into the deep learning models for iteration to minimize the loss function. This algorithm was used to classify images obtained by CLE verified as glioblastoma, brain metastases or meningioma.

### Image Interpretation by Neurosurgeons

For manual and manual/deep learning combined assessment of CLE images we created a training set, a manual test set, and a ResNet test set, each containing a total of 90 images of balanced proportions of glioblastomas, meningiomas, and metastases. Images containing obvious artifacts (*e.g.* motion artifacts) were excluded previously. The ResNet test set contained only images rated by the ResNet with an output level of 0.99 or higher. In the training set, information about the corresponding histopathologic diagnosis was available for each image, and three experienced neurosurgeons underwent training for CLE image interpretation. Subsequently, images of the two test sets were reviewed by the neurosurgeons in a blinded fashion.

### Statistical Analysis

Statistical analysis was performed using GraphPad Prism 8.3.0 (GraphPad Software, San Diego, CA). The overall predictive value was analyzed by area under the receiver operating characteristic curve (AUROC), precision-recall curve, and macro-averaged F1 analysis. For test set accuracy, comparative analysis of deep learning models and neuro-oncological surgeons, the Mann–Whitney test was used. We calculated the accuracies of the three neuro-oncological surgeons using the ratio between the number of correct diagnosed and total CLE images.

## Results

To test the hypothesis that CLE could provide an alternative method for intraoperative frozen section histology and facilitate targeted biopsy, we collected surgical specimens from 25 patients and acquired a total of 19,422 images (glioblastoma 5,668, brain metastases 6,814, meningioma 6,960) for the evaluation (**Table 1**). The specimens were either stained with fluorescein dye or nuclear green dye with similar distributed data sets of the three classes. We acquired CLE images of fresh tissue samples, which were free of freezing and additional sectioning artifacts and therefore provided well-preserved tissue architecture. More importantly, fresh tissue imaging mimics intraoperative imaging without complex and time-consuming sample processing (**Supplementary Figure 1**). Representative *ex vivo* CLE images of the three groups are shown in **Supplementary Figure 2**. All glioblastoma *ex vivo* specimens CLE displayed stellate, bright spots.

**Table 1:** Patients’ characteristics and data composition.

	Training	Test
**Total patients**	**22**	**3**
**Glioblastoma**	**7**	**1**
**Brain metastases**	**7**	**1**
**Meningioma**	**8**	**1**
**Images (fluorescein dye)**	**12,273**	**1,699**
**Images (nuclear green dye)**	**7,151**	**1,291**

Values represent patient numbers for glioblastoma, brain metastasis and meningioma and total image numbers for fluorescein and nuclear green, respectively.

### Differentiation of Malignant Tumors

**Figure 1:**
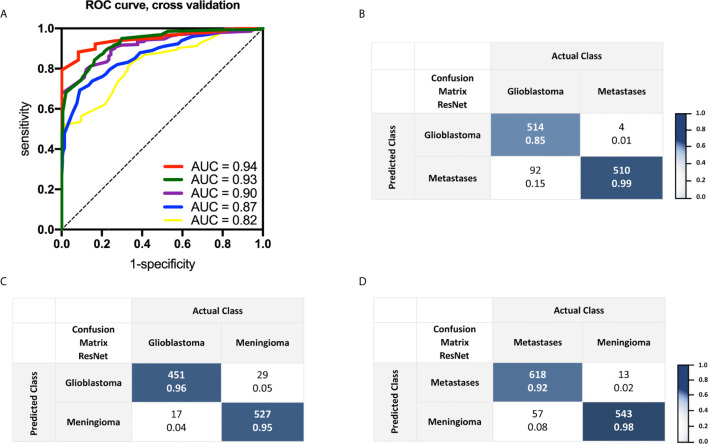
**(A)** Receiver operating curve analysis and corresponding area under the curve values received of a trained two-class residual convolutional network with five-fold cross validation. Images were obtained from seven patients with glioblastoma and brain metastases, respectively. **(B)** Confusion matrix shows outputs and actual labeling for binary classification of an additional test set, including one patient with glioblastoma and another one with brain metastases. **(C, D)** Confusion matrices indicating binary classification results of a test set for glioblastoma and meningioma as well as brain metastases and meningioma, respectively. In each cell, the number above is the count and the number below the normalized count. Images were obtained with CLE following topical *ex vivo* staining with fluorescein dye.

Conventional magnetic resonance imaging ultimately fails to distinguish between glioblastoma and brain metastases among malignant brain tumors to date. We built a residual network (ResNet18) model for binary classification to demonstrate its diagnostic capability on these highly heterogeneous tumor specimens stained with fluorescein. Specimens from eight patients for each tumor type were used (4,361 images of glioblastoma, 3,503 images of brain metastases). We evaluated CNN-based methods using a leave-one-patient out cross-validation, *i.e.* one patient always represented the test data and all others the training data. This way, inherent correlation within the image sequences did not play a role in the analysis. Receiver operating characteristic curves, corresponding AUC values, and confusion matrix results are shown in **Figures 1A, B**. An average accuracy of 90.9% was achieved following five-fold cross validation. Since fluorescein based CLE imaging is susceptible to artifacts and includes a significant amount of non-diagnostic images, automatic approaches are required to filter relevant data for diagnosis. After we applied a threshold for the output level of the test data of 0.999, the accuracy improved to 100% in this subset (**Table 2**). 35.6% of the images in the test set had an output level of 0.999 or higher, thus providing a potential filter for non-diagnostic images. Next, binary classification for glioblastoma and meningioma as well as brain metastases and meningioma were performed (**Figures 1C, D**). In a test set containing image data of 1,024 images showing glioblastoma and meningioma CLE images, an overall accuracy of 95.5% was achieved. ResNet18 showed 94.3% accuracy for differentiation of brain metastases and meningioma in a test set containing 1,231 CLE images.

**Table 2:** Accuracy, macro-averaged F1 and area under the curve analysis of trained networks for multiclass classification (glioblastoma, brain metastasis, and meningioma) and binary classification (glioblastoma and brain metastasis).

Network	Accuracy (%)	F1/AUC	Rate of diagnostic images (%)
ResNet18			
Test total	85.8	92.3% (F1)	32.3
Test confidence > 0.999	93.6		
ResNet18 filtered			
Test total	87.3	93.2% (F1)	36.3
Test confidence > 0.999	98.6		
InceptionNet			
Test total	82.9	90.6% (F1)	17
Test confidence > 0.999	91.1		
			
ResNet18 binary classification			
Test total	90.9	0.92 (AUC)	35.6
Test confidence > 0.999	100		

ResNet filtered contained manually selected data free of substantial artifacts. The ratio of images rated with an output level of 0.999 or higher and the amount of total images are indicated as diagnostic images. Images were obtained with CLE following topical ex vivo staining with fluorescein dye. Macro-averaged F1 was calculated using the following equation: 2*(precision_m_*recall_m_)/(precision_m_+recall_m_).

### Multiclass Classification With ResNet18 and InceptionNet

For practical reasons, further multiclass classification of common brain neoplasms is needed. Subsequently, we employed a residual network (ResNet18) model and an Inception network (InceptionNet) model to assist the diagnosis of brain tumor tissues including meningiomas based on CLE image data. To train and test the ResNet18 and InceptionNet models, we incorporated CLE images from 25 patients and labeled them as “glioblastoma”(eight patients, 5,322 images), “metastasis” (eight patients, 4,120 images) and “meningioma” (nine patients, 4,529 images), respectively.

**Figure 2:**
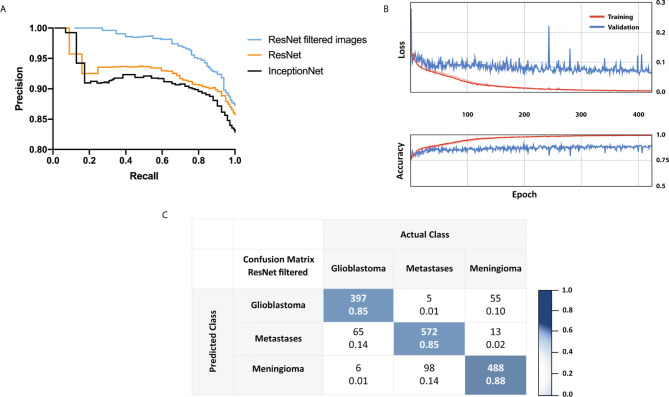
**(A)** Test set results with Precision-Recall curves received for a trained three-class residual convolutional network, Inception network and a trained residual network with manually selected images. In **(A)** 12,273 images were used for training and 1,699 for the test set. In the preselected data set, 5,261 images were found suitable for training, and 647 were available for the test set. **(B)** Five-fold cross validation results. **(C)** Confusion matrix showing ResNet18 outputs and labeling of a test set using preselected images after images including substantial artifacts were manually removed. Images were obtained with CLE following topical *ex vivo* staining with fluorescein dye. In each cell, the number above is the count and the number below the normalized count.

The precision-recall curve analysis of concurrent three-class tumor-type prediction showed macro-averaged F1 values of up to 0.92 (**Figure 2**). Importantly, five-fold cross validation confirmed the networks’ validity. InceptionNet showed no superior performance with a macro-averaged F1 score of 0.91. ResNet18’s overall accuracy of 85.8% for three-class prediction was slightly improved by manually filtering CLE images before employing the ResNet18 model. Considering the network’s confidence for the assessment of CLE images provides a possible feasibility for analysis, regarding the lack of histopathological information in most images. Therefore, we calculated the rate of diagnostic images with high confidence as ratio of images with output levels of 0.999 or higher and the overall amount of images. Results for both the deep learning models and the ResNet18 model with preselected data only are summarized in **Table 2**. Test accuracy was 93.6% when a confidence level of >0.999 was applied. 548 of 1,576 (32.3%) images were marked with a confidence of 0.999 or higher. A ResNet18 model trained and tested with preselected data had an accuracy in the test set of 87.3 and 98.6% for images with a confidence level >0.999. Here 36.7% (355/966) of images had an output level of 0.999 or higher. Test set accuracies for individual classes of glioblastoma, brain metastases, meningioma and the respective rate of diagnostic image data are presented in **Table 3**.

**Table 3:** Accuracy analysis of residual neural network with multiclass classification for individual classes.

Class/Network	Accuracy ResNet18 unfiltered	Accuracy ResNet18 unfiltered with threshold >0.999	Rate of diagnostic images
Glioblastoma ResNet18 unfiltered	92.3	93.8	57.5
Metastases ResNet18 unfiltered	89.3	94	20
Meningioma ResNet18 unfiltered	89.9	99.5	25.9
Glioblastoma ResNet18 filtered images only	92.3	98.6	37.8
Metastases ResNet18 filtered images only	93.5	100	53.3
Meningioma ResNet18 filtered images only	88.2	98.6	5.5

The ratio of images rated with an output level of 0.999 or higher and the overall images are indicated as diagnostic images. ResNet18 filtered contained manually selected data free of substantial artifacts. A threshold of >0.999 indicates only images with output values of 0.999 or higher as rated by the network were included in the analysis. Images were obtained with CLE following topical ex vivo staining with fluorescein dye. Percentage values are indicated.

### Complementing Manual With Automated Analysis

**Figure 3:**
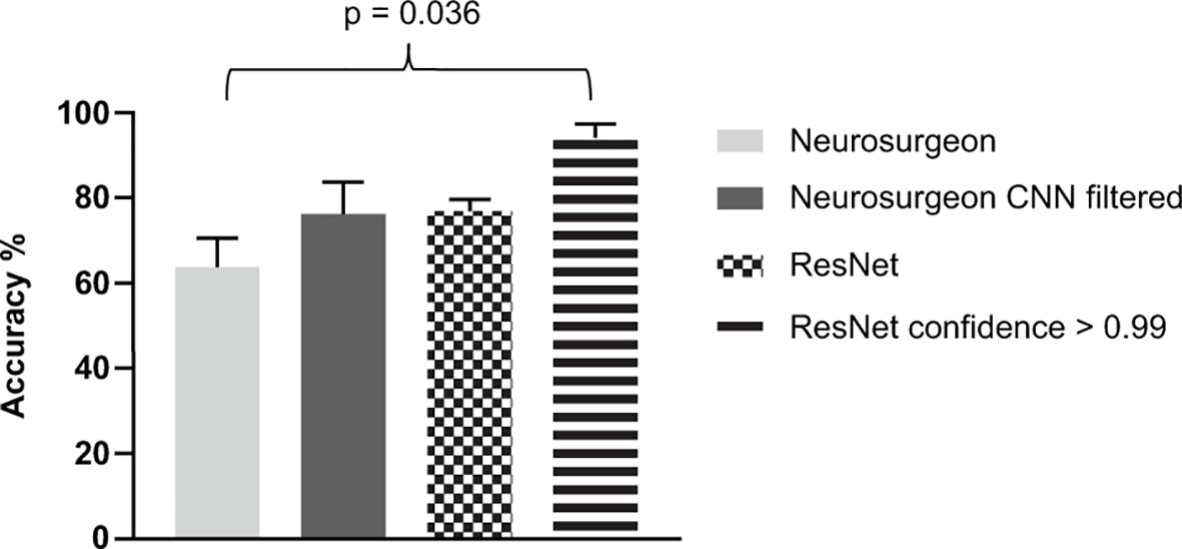
Comparative analysis of manual, deep learning based and combined assessment of glioblastoma, brain metastasis and meningioma images obtained by confocal laser endomicroscopy. Balanced test sets containing 90 images were anonymized and evaluated by trained neuro-oncological surgeons. Results are displayed in the first column. A second test set was evaluated by the surgeons including images rated by the residual network with a confidence of 0.99 or higher (2nd column). Overall accuracies of residual network test set analysis and results of the image subgroup rated with an output level of 0.99 or higher are displayed. Average accuracy of at least three independent experiments and five-fold cross validation are shown, respectively.

Since CLE accompanies a high amount of artifacts and potential non-diagnostic images, we had a close look at the output levels, also described as network’s confidence for single image analysis. When we analyzed images with output levels of 0.99 or higher, average accuracies of 92.3% were achieved applying ResNet18 for multiclass classification of CLE data. Independent analysis of two neuro-oncological surgeons of these images showed abundant histopathological data compared to the entire data set. Since CLE images are almost immediately displayed during surgery, we wondered if a combination of expert opinion and real-time automated analysis would facilitate decision making. We compared neurosurgical assessment of balanced data sets, containing equal numbers of glioblastoma, brain metastases, and meningioma images to CNN performance (**Figure 3**). There was a tendency towards higher accuracy when images were selected by CNN’s output level compared to manually selected images, lacking artifacts or low contrast. However, neuro-oncological surgeons could not achieve accuracies of ResNet18 image analysis rated with an output level of 0.99 or higher.

### Convolutional Neural Network for Cell Density and Nuclear Analysis

**Figure 4:**
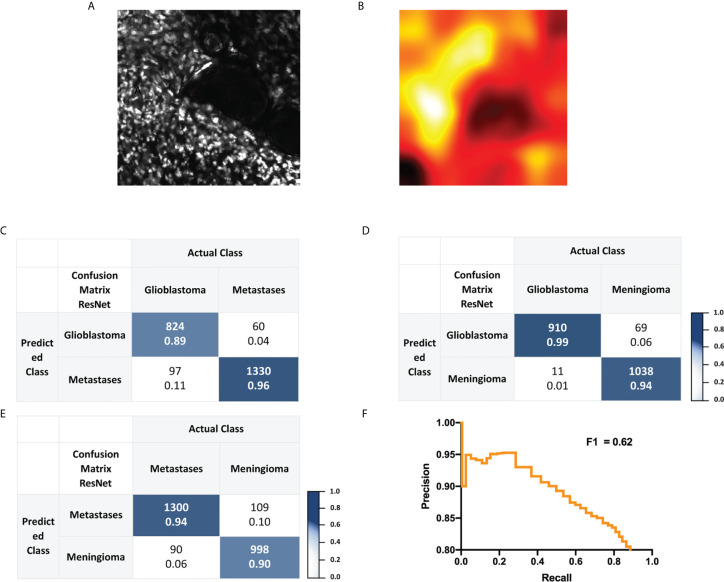
**(A, B)** Representative glioblastoma CLE image following topical staining with nuclear green and corresponding heat map. **(C–E)** Confusion matrix for residual network based binary classification. Images were labeled as “glioblastoma”, “brain metastasis” or “meningioma” and were obtained with CLE following topical *ex vivo* staining with nuclear green dye. In each cell, the number above is the count and the number below the normalized count. **(F)** Precision-Recall curve analysis and corresponding macro-averaged F1 score received for a test set of a trained three-class residual network.

Staining with nuclear green dye offers a readout for cellular density. CLE images of resected tissue samples stained with nuclear green resulted in image sets with consistently high quality and strong nuclear staining (**Figure 4A**). Exact cell count and morphology might therefore result in sufficient CNN performance (**Figure 4B**). Analysis for binary classification of nuclear green images is presented in **Figures 4C–E** and in **Figure 4F** for multiclass classification. A macro-averaged F1 score of 62% was achieved for classification of the three tumor types. When a threshold of confidence of 0.999 was applied, 124 of 2,990 images were suitable, and an accuracy of 94% resulted in the test set.

**Figure 5:**
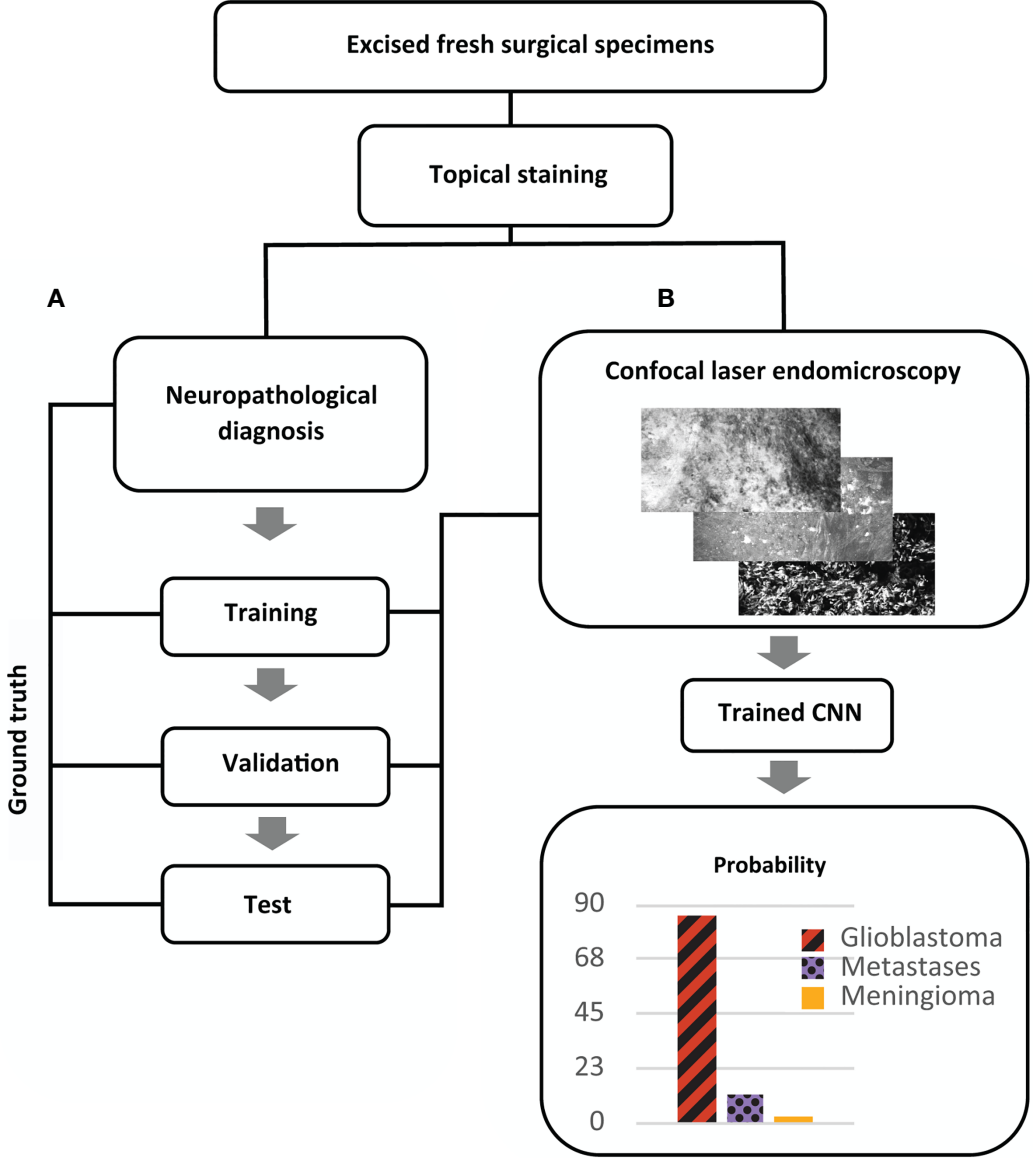
Proposed pipeline for intraoperative diagnosis of brain tumors with confocal laser endomicroscopy after image acquisition, training and test phase of a convolutional neural network (CNN). **(A)** Experimental design for construction and validation of a CNN for multiclass classification of brain tumors. Fresh specimens are stained with fluorescein dye, and images are acquired with confocal laser endomicroscopy (CLE) by a single user. Afterwards the specimens undergo histopathological diagnostics (as described in *Materials and Methods*) providing ground truth for training and validation of the CNN. CLE contained a portable hand held probe and a touch screen. **(B)** Schematic workflow for *ex vivo* brain tumor diagnosis providing CLE images with an additional confidence level. By applying a confidence threshold, non-diagnostic images are not considered for evaluation.

Following validation of the neural network models, we created a model for on-site tumor diagnosis of glioblastoma, brain metastases and meningioma. We propose a final evaluation of CLE imaging by the neurosurgeon with the aid of the networks’ output levels to estimate diagnostic probability. The output levels do not display percentage of diagnostic probability. Schematic network construction and the proposed streamlined workflow with tissue to diagnose pipeline are shown in **Figure 5**.

## Discussion

The neurosurgical workflow and surgical performance could benefit from CLE in at least two aspects. First, its use could substitute for conventional frozen section pathology. Second, it could be used to confirm completeness of the resection by scanning the walls of the resection cavity.

CLE imaging combined with Computer Aided Diagnosis (CAD) successfully predicted the three most common encountered brain tumor entities. Manual preselection of the data showed marginal effect on network accuracy and the rate of diagnostic images. However, there was a limited and probably too small amount of data left for training, affecting the overall output. Further, the selection by a neurosurgeon, focusing on known structures and contrast, might not correlate with the network’s criteria used for the output level. Of note, output levels were higher for brain metastases and glioblastoma for two and three-class networks as compared to meningioma. We suspect that the high predictive power of glioblastoma CLE images is due to the characteristic bright spots occurring almost exceptional in glioblastoma specimens. Primarily noted from *ex vivo* imaging, some cells might uptake fluorescein following prolonged exposure to the dye or *via* influx of dye into damaged cells (17). Interestingly, outputs were rather less predictive when nuclear green dye was used. This suggests that in case of fluorescein application other features than nuclear density were chosen.

Diagnostic sensitivity of CLE has been previously described between 52.97 and 90% in case of low grade and high grade gliomas and meningioma *ex vivo* specimens analyzed by neuropathologists and neurosurgeons. Diagnostic sensitivity was only 37% for brain metastases (18, 19). Confocal scanning microscopy allows rapid histopathological assessment for a variety of brain neoplasms including, gliomas, metastases, and pituitary tumors faster than conventional frozen section (20). However, sifting manually through the images is tedious and impractical for high throughput imaging. Therefore, an upstream network is essential for filtering non-diagnostic images in a fast and feasible manner (21).

The high predictive potential of our network for brain metastases might present an integral part of fully automated diagnosis in the near future, complementing the model with preoperatively diagnosed metastases *via* computer-aided detection in magnetic resonance imaging (22, 23). Izadyyazdanabadi et al. showed promising results applying neural network models to categorize CLE data of brain neoplasms into diagnostic and non-diagnostic images, though not specifying the actual tumor entity (24).

For other fields like Barrett’s esophagus, CLE combined with automated image processing approaches has shown not only thorough diagnostic potential, but also decrease of biopsy samples and the ability to classify the pathology (25). CAD of CLE based data also showed promising results in diagnosing inflammatory bowel disease and discriminating neoplastic *versus* non-neoplastic epithelium in head and neck cancer (26, 27). Similar deep networks were used to evaluate cancerous colon tissue following CLE imaging (28).

Kamen et al. early described the use of an automated tissue differentiation algorithm with machine learning in order to classify CLE images of glioblastomas and meningiomas. However, the clinical impact distinguishing solely these tumor types is limited and state-of-the-art CNNs for classification were not used, which resulted in an average accuracy of 84% (29). When aiming for automated tumor diagnosis by CLE image analysis in neurosurgery, multiclass networks need to be designed and trained for multiple tumor types, reactive and normal brain tissues. To our knowledge, such a multiclass classifier has not been applied for CLE image analysis, and prior studies have not distinguished glioblastoma from cerebral metastases CLE images with machine learning.

We believe CLE based automated data processing is less expensive and more efficient than the current technique, even if time needed for data transfer and consultation with a neuropathologist are included into that consideration. Conventional workflows without automated approaches necessitate a functional network for data transfer and communication, trained and available neuropathologists for image interpretation, time to review single images, and constant technical support. Each step represents a potential economic and technical barrier, while computational costs are limited. Further, the proposed pipeline for *ex vivo* diagnosis is an alternative for neurosurgeons where diagnosis by neuropathologists is not broadly available. The use of CLE-assisted fluorescent surgery not only is an improvement of immediate histological diagnosis as compared to time-consuming hematoxylin and eosin staining, but could also improve representation of the borders of tumor and normal tissues (30).

However, caution is advised in using the networks’ confidence for single image analysis. In our opinion, confidence levels provide an aid for intraoperative expert analysis. Multiple images and affiliated output levels should be evaluated in the same region. Further, we acknowledge several limitations to our study. Our model cannot readily be applied to clinical situations yet, unless training for additional tumor types and normal as well as non-tumor mimickers is completed. The current model might be applicable for patients who have high likelihood of glioblastoma, cerebral metastases, or grade I meningioma based on standard radiographic evaluation. The feasibility of CLE-assisted multifluorescent surgery also has to be increased by extending the usability to other fluorescent agents like 5-aminolevulinic acid (30). In order to provide high-quality data *in vivo*, the kinetics of the fluorophore agents and administration techniques have to be taken into account. Furthermore, *in vivo* validation is mandatory, since the study did not utilize intravenous fluorescein application which potentially allows a more homogenous staining than topical application, however also underlies decreasing fluorescence signal over time after injection (31). Quality of *in vivo* acquired images is limited by the fluorophore kinetics as some dyes, in particular fluorescein, washes out leading to low contrasted tissue if image acquisition is delayed. In contrast, e*x vivo* imaging after topical staining is significantly less affected by the timing of fluorescein administration. Insufficient contrast can be avoided by readministration of fluorescein and thus increase image quality (32). Future analysis will include an assessment of tumor microvasculature in addition to tumor cell morphology and architecture. Interpretation of erythrocyte flow, thrombosis, and velocity changes may help to classify glioma subtypes, normal and injured brain tissues (33). Due to the lack of blood flow in *ex vivo* samples, these features were not included during the CNN training process in this study.

We continue to enlarge our sample size and anticipate to extend the model’s labeling ability when a larger data set with different tumor entities will be available. In addition, expert’s diagnostic recognition may be improved using machine learning algorithms. For example, Izadyyazdanabadi M. et al. used image style transfer method based on permanent hematoxylin and eosin staining and therefore enhanced diagnostic quality of glioma CLE images (34). Among other factors, colorization of the images provided advantages for the analysis. Therefore image style transfer is a promising tool, which could be integrated into the described workflow for on-the-fly interpretation of CLE images and should also be tested in brain metastases. Additionally, a crucial point for future real-time diagnosis is the automated reduction of artifacts affecting the analysis. Therefore, the use of transfer learning from intermediate endpoints may overcome non-diagnostic image sections and thus improve accuracy as described by Aubreville et al. (35).

## Conclusions

The use of machine-learning algorithms following CLE imaging achieved high accuracy in the prediction of three different brain tumor types when output levels were assessed. The developed algorithm enables CLE to be integrated into the clinical workflow as a tool for almost real-time tissue diagnosis. Beyond simply gaining an orientation about tumor entity, fast high-volume image processing facilitates a high amount of artifact-free digital biopsies. Thereby, intra-tumor heterogeneity and information about resection margins can be taken into account when performing tumor resection or planning postoperative radiation. Further investigations to improve overall performance are needed before the method can become part of neurosurgical routine.

## Conflict of Interest

DS was employed by the company Clevertech Inc.

The remaining authors declare that the research was conducted in the absence of any commercial or financial relationships that could be construed as a potential conflict of interest.

## Author's Note

Portions of this work were presented in abstract form at the 2019 Computer Assisted Radiology and Surgery Conference, Rennes, France, June 20, 2019.

## Data Availability Statement

The data associated with the paper are not publicly available but are available from the corresponding author on reasonable request.

## Ethics Statement

The studies involving human participants were reviewed and approved by the Ethics Committee II, University of Heidelberg, Medical Faculty Mannheim. The patients provided their written informed consent to participate in this study.

## Author Contributions

VR, FE, and AZ performed experiments. DS and AZ analyzed results and prepared figures. FE and DH designed the research. AV provided histopathological analysis, AZ and FE drafted the paper, and all authors reviewed and commented on the report. All authors contributed to the article and approved the submitted version.
