# Supplementary material for: Deep Neural Network for Differentiation of Brain Tumor Tissue Displayed by Confocal Laser Endomicroscopy
Source: Front Oncol. 2021 May 11;11:668273. doi: 10.3389/fonc.2021.668273 (PMC8147727; doi:10.3389/fonc.2021.668273)
Supplement: Supplementary file 1 [file DataSheet_1.docx]

Supplementary Material


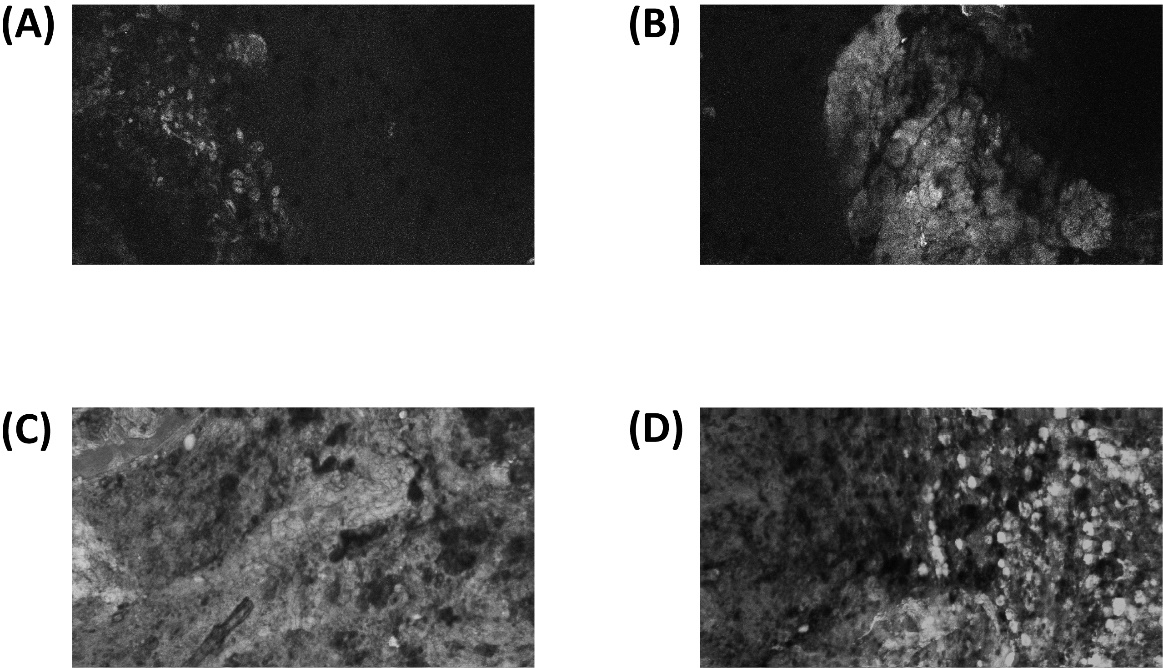


**Supplementary Figure 1.** Representative *in vivo* confocal laser endomicroscopy imaging of glioblastoma with intravenous fluorescein application (**A**, **B**) and *ex vivo* imaging following glioblastoma resection applying topical staining with fluorescein dye (**C**,**D**).


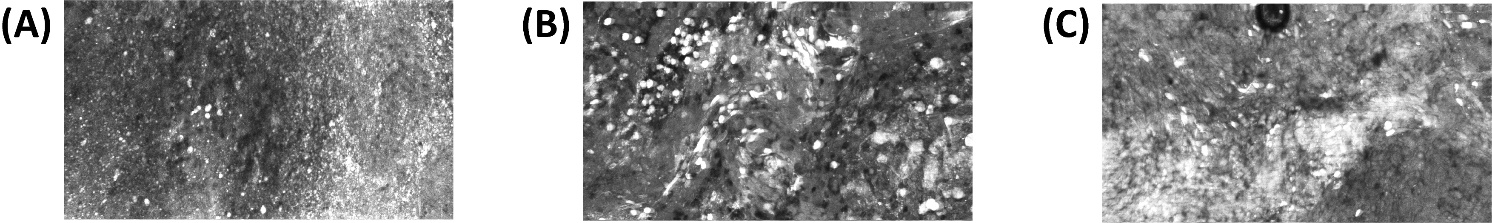


**Supplementary Figure 2.** Representative *ex vivo* images of the three tumor types glioblastoma (**A**) brain metastasis of non-small cell lung cancer (**B**) and meningioma WHO grade I (**C**) obtained by confocal laser endomicroscopy after topical staining with fluorescein dye. Confocal laserendomicroscopy of glioblastoma tissue shows characteristic bright spots and hypercellularity. Brain metastasis displays hypercellularity and atypical cells, while meningioma reveals similar features to conventional histopathology including whirls and psammoma bodies.
